# Unraveling dynamics of nuclear pore and chromatin via HS-AFM

**DOI:** 10.1007/s12565-025-00849-y

**Published:** 2025-05-19

**Authors:** Goro Nishide, Richard W. Wong

**Affiliations:** 1 Division of Nano Life Science, Graduate School of Frontier Science Initiative, Kanazawa University, Kanazawa, 920-1192 Japan; 2 WPI Nano Life Science Institute (WPI-NanoLSI), and Institute for Frontier Science Initiative, Kanazawa University, Kakuma-Machi, Kanazawa, 920-1192 Japan

**Keywords:** Spider, Cobweb, Coil-assembly-rod-doughnut (CARD), Ligand-Induced Dimerization (LID), Nuclear pore complex (NPC), High Speed Atomic Force Microscopy (HS-AFM), ORF6

## Abstract

High-speed atomic force microscopy (HS-AFM) enables real-time visualization of biological processes with nanometer-level resolution. This review highlights how HS-AFM has been instrumental in uncovering the dynamic interplay between nuclear pore complexes (NPCs)—which regulate nucleocytoplasmic transport—and genome guardians, including DNA repair proteins and chromatin regulators. Structurally, the NPCs resemble a multi-layered spider cobweb, serving as crucial molecular gatekeepers for maintaining cellular homeostasis, while genome guardians safeguard genomic integrity through DNA repair and chromatin organization. Through HS-AFM, the researchers have gained unprecedented insights into NPC dynamics, revealing their adaptability during nuclear transport, chromatin reorganization, and viral infection. It has also elucidated how genome guardians interact with NPCs, influencing chromatin organization at the nuclear periphery and regulating nucleocytoplasmic trafficking. These discoveries underscore the critical role of NPC-genome interactions in genome stability, gene expression, and nuclear transport, with broad implications for diseases such as cancer, viral infections, and neurodegenerative disorders. In conclusion, HS-AFM has transformed our ability to study the nuclear landscape at the nanoscale, bridging the gap between structural biology and functional genomics. By capturing the real-time molecular dynamics of NPCs and chromatin, HS-AFM provides an essential tool for unraveling the mechanisms that govern nuclear transport and genome regulation. Future advancements in HS-AFM technology, including higher temporal resolution, correlative imaging, and AI-driven analysis, will further expand its potential in biomedical research, paving the way for novel diagnostic and therapeutic strategies.

## Atomic force microscopy (AFM)

Atomic force microscopy (AFM), initially developed for use on samples in a vacuum (Binnig et al. 1986), is a high-resolution technique that uses a probe tip attached to a microcantilever to scan sample surfaces. As the probe interacts with the sample, the resulting forces—electrostatic, van der Waals, and capillary—are measured through force spectroscopy, allowing detailed analysis of physical properties. AFM’s capability to map surfaces at the nanometer scale offers a unique advantage over techniques like transmission electron microscopy (TEM), enabling the exploration of protein arrangements within viral membranes.

AFM, TEM, scanning electron microscopy (SEM), and focused ion beam scanning electron microscopy (FIB-SEM) are powerful imaging techniques, each with unique advantages and limitations for studying surface structures. AFM is particularly well-suited for high-resolution surface imaging at the nanometer scale without requiring sample staining or a vacuum environment. Unlike TEM, which provides detailed internal structure visualization by transmitting electrons through ultra-thin samples, AFM operates in ambient or liquid conditions, making it highly suitable for imaging biological specimens in near-physiological states. Compared to SEM, which captures detailed surface topography by scanning a sample with an electron beam, AFM offers higher vertical resolution and the ability to measure mechanical properties, such as stiffness and adhesion forces, at the molecular level. FIB-SEM, which combines SEM with a focused ion beam for precise cross-sectioning, enables 3D reconstruction of complex structures, but it requires extensive sample preparation and is often destructive. In contrast, AFM provides non-destructive imaging, allowing repeated measurements of delicate biological samples over time. While TEM and SEM excel in high-throughput imaging of fixed and coated samples, AFM is uniquely advantageous for studying dynamic biological processes and nanoscale surface interactions in real time.

Soon after its inception, AFM demonstrated its ability to image samples in liquid environments (Fukuma 2020a), making it a valuable tool in biological research. This versatility allows AFM to produce highly detailed, three-dimensional maps without the need for chemical staining or labeling (Fukuma 2020b), a significant benefit in the study of biological specimens. Among the most advanced variations of AFM is three-dimensional AFM (3D-AFM), which maps forces in all three dimensions to visually represent molecular distributions (Fukuma and Garcia 2018). Initially used for analyzing solvent molecules on substrates, 3D-AFM is now employed to visualize the internal structure of biological cells (Penedo et al. 2021).

Recent innovations in AFM have expanded its use to biopolymers—key biomolecules like DNA and proteins. Sumikama et al. (2022) developed a technique for generating 3D-AFM images of biopolymers, leveraging the bead-spring polymer model, a supra-coarse-grained simulation, to approximate chromosomes and probe interactions(Sumikama et al. 2022).

## High-speed AFM (HS-AFM)

While conventional AFM has provided valuable insights, its temporal resolution is limited. Traditional AFM takes about 30 s to capture a single image, which is too slow to observe rapid biological processes. To address this limitation, high-speed atomic force microscopy (HS-AFM) was developed, enhancing the temporal resolution to capture dynamic behaviors in biological samples(Ando et al. 2001). Modern HS-AFM systems can take images in as little as 100 ms, enabling precise visualization of molecular movements and dynamic processes in real time. Despite the rapid interactions between the probe and the sample, HS-AFM is designed to be minimally invasive, preserving the structural and dynamic integrity of the sample during extended imaging(Umeda et al. 2023; McArthur et al. 2025; Shibata et al. 2017) (Table 1).

**Table 1:** comparing conventional AFM, 3D-AFM, and HS-AFM, emphasizing the advantages of HS-AFM

Feature	Initial AFM	3D-AFM	HS-AFM (High-Speed AFM)
Imaging speed	Minutes to hours per frame	Slower than HS-AFM, but allows multi-angle imaging	Milliseconds per frame (real-time dynamics)
Dimensionality	2D (topography)	3D reconstruction of structures	2D, but can capture dynamic 3D conformational changes over time
Spatial resolution	High (sub-nanometer)	High (sub-nanometer)	High (sub-nanometer)
Temporal resolution	Poor (limited by scan speed)	Improved but still slow	Excellent (sub-100 ms/frame)
Sample compatibility	Requires stable samples, often in air or liquid	Can probe different depths	Ideal for soft biological molecules in physiological conditions
Application scope	Static imaging of surfaces	Structural studies with depth profiling	Real-time observation of molecular dynamics
Biological relevance	Limited (slow scan can introduce artifacts)	Better structural insights	Captures molecular interactions in near-native conditions
Key advantages	High-resolution surface imaging	3D information	Fast, real-time imaging of dynamic biomolecules
Key limitations	Very slow, artifacts from drift	Trade-off between resolution and depth scanning	Requires specialized setup, delicate cantilevers

HS-AFM achieves rapid imaging without damaging samples through several key innovations. First, it employs small, fast cantilevers with low stiffness and high resonance frequency, which reduces the risk of disturbing delicate biological samples. Additionally, HS-AFM utilizes precision laser detection via a specialized optical beam deflection system, allowing for accurate measurement of cantilever movements. A high-speed feedback system further enhances the technique by ensuring that the probe maintains the correct distance from the sample, even during rapid scanning. This is complemented by high-speed data acquisition and processing, which enables the creation of accurate images that capture dynamic biological behaviors in real-time. To effectively use HS-AFM for biological materials, it is essential to balance achieving sufficient temporal resolution while minimizing invasiveness to avoid altering or damaging the sample (Umeda et al. 2023).

HS-AFM has proven to be a powerful tool for visualizing dynamic changes and functional processes in a wide range of biomolecular systems (Ganser and Uchihashi 2024). One of its major applications is in the study of transmembrane and peripheral membrane proteins, where it captures real-time interactions with cell membranes (Jiang et al. 2024; Pan et al. 2023). HS-AFM has also been instrumental in observing cytoskeletal structures and their interactions with regulatory proteins, providing crucial insights into cellular dynamics (Flechsig and Ando 2023). Furthermore, it has advanced our understanding of nucleic acids, such as DNA and RNA, by enabling the study of their interactions with binding proteins—an essential aspect of gene expression and regulation (Suzuki et al. 2015). Representing a major advancement in biological imaging, HS-AFM allows the observation of molecular structures and processes at nanometer resolution in near real-time (Umeda et al. 2023). Unlike conventional AFM, HS-AFM enables the visualization of molecular movements and interactions as they occur, making it particularly suited for studying complex and rapid processes within cells (Sajidah et al. 2024). This capability has been especially beneficial for exploring dynamic interactions such as viral protein docking (Lim et al. 2020a, 2023, 2021, 2020b; Nishide et al. 2023), extracellular vesicles (Sajidah et al. 2022), and nuclear pore complexes and chromatin within the nucleus.

HS-AFM has been applied to the investigation of intrinsically disordered proteins, which lack fixed structures and exhibit dynamic behavior (Kodera et al. 2021; Ando 2022; Sanganna Gari et al. 2023). Additionally, it offers a window into the function of various molecular interactions (Sumino et al. 2024; Marchesi et al. 2021; Takeda et al. 2023) and the formation of amyloid fibers, which are associated with diseases like Alzheimer’s(Watanabe-Nakayama et al. 2016). Beyond these biological structures, HS-AFM has facilitated the study of membrane vesicles(Sajidah et al. 2022) and membraneless organelles, helping researchers probe the structure and function of cellular components that do not have surrounding membranes (for example, nuclear pores). It has even extended into the realm of artificial molecules, allowing scientists to explore the dynamic properties of synthetic structures. Collectively, these applications highlight HS-AFM’s capability to unlock new opportunities for studying biomolecular systems and comprehending their dynamic and mechanistic properties (Ando et al. 2023).

**Fig. 1:**
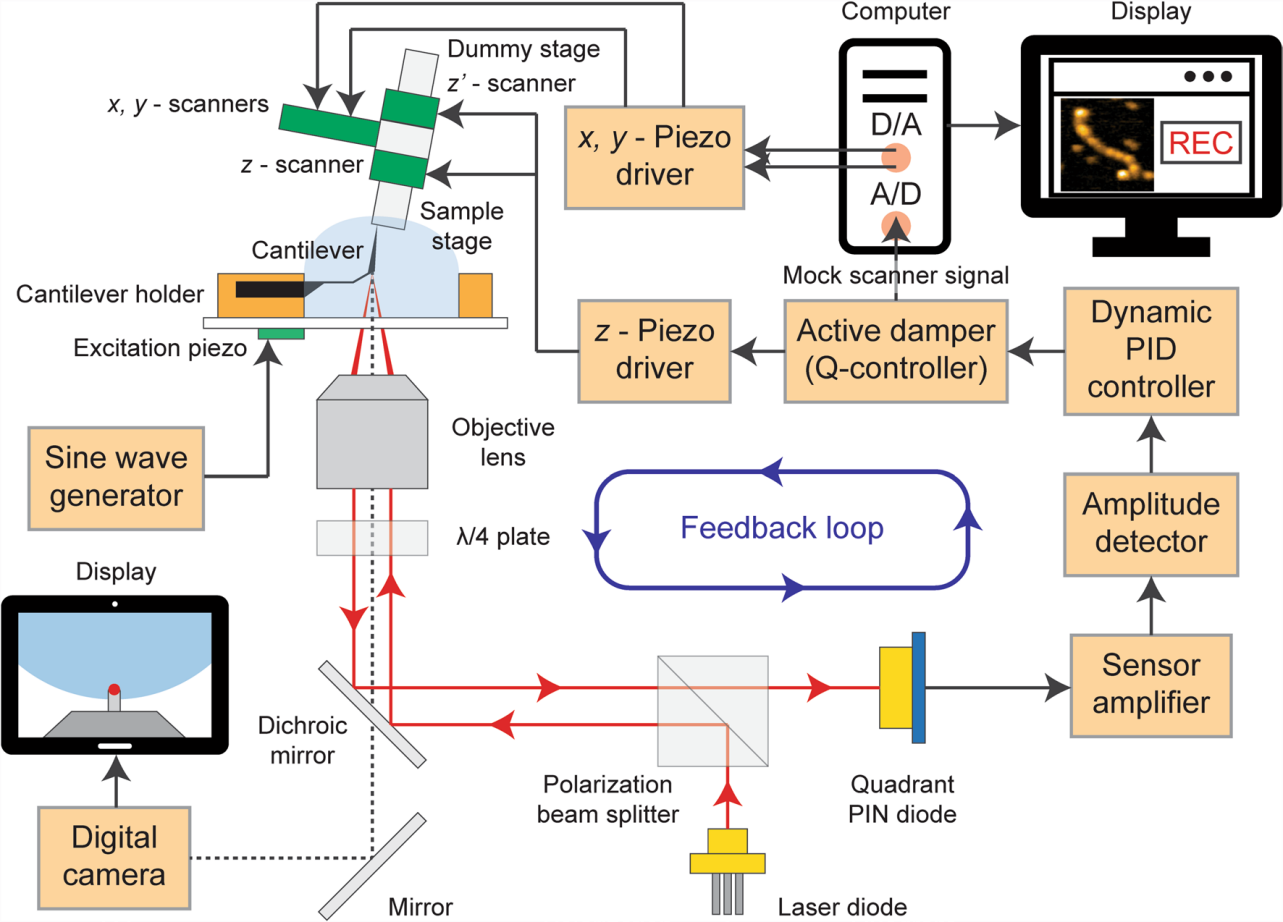
Schematic of HS-AFM system. This schematic depicts a HS-AFM setup integrated with a custom inverted optical microscope. The system features a long-working distance objective lens, part of the optical beam deflection (OBD) detector, used to observe the cantilever and sample stage through a digital camera. A glass slide with a cantilever holder and liquid cell is placed on the microscope stage. The cantilever chip is mounted with its tip facing upwards, while the sample stage, mounted on a Z scanner and positioned above the cantilever, faces downwards. A laser beam is focused on the cantilever, and the reflected light is directed to a photodiode. A quarter-wavelength plate and polarized beam splitter separate the incident and reflected beams. The cantilever vibrates at its resonance frequency in a buffer solution, with the vibration amplitude stabilized through feedback control. The Z scanner employs a counterbalance to minimize vibration artifacts. X and Y scan signals are generated via a DA converter, while an active Q control damper records sample height via an AD converter. The active Q control functions similarly to the Z scanner’s displacement mechanism

HS-AFM operates in “tapping mode,” also known as amplitude modulation or intermittent contact mode. This mode is particularly useful for studying sensitive biological materials because it gently taps the sample surface, reducing the risk of damage. HS-AFM is designed for high-speed imaging, with a setup similar to traditional AFM but optimized for speed. The system continuously adjusts the position of the sample stage in the vertical (Z) direction to maintain consistent tapping force, allowing for the creation of detailed topographic images (Fig. 1) (Ando et al. 2023).

Despite its numerous advantages, HS-AFM has several limitations that researchers must consider. One of the primary challenges is its limited imaging depth, as HS-AFM is most effective for studying surface structures and cannot easily capture subsurface or deeply embedded biomolecular interactions. Additionally, sample preparation can be complex and delicate, requiring careful immobilization of soft biological materials to prevent movement during imaging. Another drawback is potential tip-induced artifacts, where the AFM probe itself may influence or alter the sample’s natural behavior, particularly for highly dynamic or fragile biomolecules. Furthermore, HS-AFM has a relatively small scanning area, making it less suitable for analyzing large cellular structures or tissues. The technique also requires specialized expertise and costly instrumentation, limiting its accessibility compared to other imaging methods such as electron microscopy. Finally, while HS-AFM provides excellent temporal resolution, it is primarily restricted to surface dynamics, making it less effective for studying complex 3D interactions within cells. Addressing these challenges will be crucial for further expanding the applications of HS-AFM in biological research.

## Structural overview of the nuclear pore complex

**Fig. 2:**
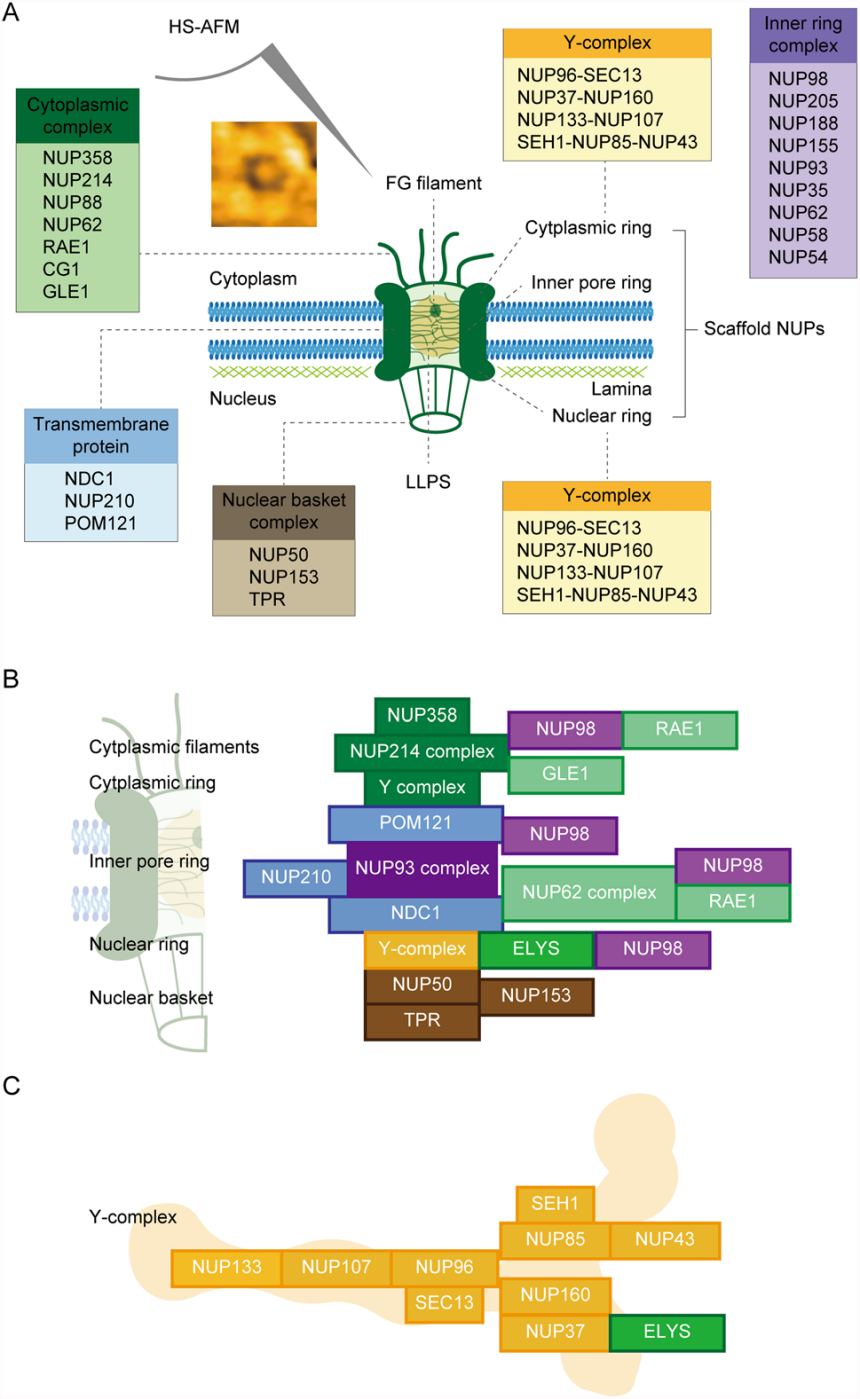
Schematic representation of nuclear pore complex (NPC) architecture. **A** The cytoplasmic complex is shown in green, transmembrane proteins in blue, the nuclear basket complex in brown, the inner ring complex in purple, and the Y-complex in yellow. **B** Detailed composition and spatial arrangement of the NPC are illustrated. **C** The relative positions of nucleoporins (NUPs) within the Y-complex are indicated

Nuclear pore complexes (NPCs) are essential for regulating the selective transport of macromolecules between the nucleus and cytoplasm, forming a complicated, multi-layered central channel, which resembles a spider web (Wong 2015, 2021; Mohamed et al. 2020, 2017). Structurally, NPCs consist of a multi-layered central channel, assembled from nucleoporins (Nups), including scaffold Nups that maintain structural integrity and phenylalanine-glycine (FG)-rich Nups, which form a selective permeability barrier (Lin and Hoelz 2019). The NPC is composed of a symmetrical central core and asymmetrical peripheral structures that form the cytoplasmic filaments (CF) and nuclear basket (NB) (Mallik et al. 2024; Raices and D’Angelo 2022) (Fig. 2A). The central core is organized into four concentric rings: an inner ring (IR), connected by linker interactions around the central channel; two outer rings on the nuclear and cytoplasmic sides (NR and CR), made up of the coat nucleoporin complex (CNC, also known as the Y-complex or Nup107–160 complex); and a luminal ring that surrounds the NPC, where pore membrane (POM) proteins anchor the outer rings to the nuclear envelope. Nups are classified based on their location within the NPC, such as transmembrane Nups in the luminal ring, scaffold components of the IR, NR, and CR, and FG (phenylalanine-glycine)-rich Nups found in the NB, CF, IR, and central pore channel (Fig. 2B and C) (Hoogenboom et al. 2021; Larizza and Colombo 2024). The way FG-Nups are organized and interact to form the selective barrier is still unclear, with several models proposed to explain the transport mechanism (Wu et al. 2025).

NPC is also a central hub for nucleocytoplasmic transport (NCT) (Yang et al. 2023), transport surveillance (Ikliptikawati et al. 2023), cell cycle regulation (Lima and Ferreira 2024; Nakano et al. 2011), gene expression (Ikliptikawati et al. 2024; Larizza and Colombo 2024), DNA repair(Gasser and Stutz 2023), epigenetic modulation(Brickner 2023), super enhancer trapping (Hazawa et al. 2024) and genome maintenance (Buxboim et al. 2023; Simon et al. 2024; Raices and D’Angelo 2022; Larizza and Colombo 2024; Wong and D’Angelo 2016).

The NPCs undergo continuous remodeling during nucleocytoplasmic transport, mitosis, and stress responses, and dysfunctions in NPC components have been implicated in cancer, viral infections, and neurodegenerative diseases (Sajidah et al. 2024; Zhang et al. 2025; Ikliptikawati et al. 2024). During mitosis, the disintegration of the nuclear membrane results in the swift degradation of NPCs, enabling some NPC proteins to assume vital functions in kinetochore architecture, spindle bipolarity, and centrosome stability(Lima and Ferreira 2024; Kutay et al. 2021; Hartono et al. 2019; Hashizume et al. 2013a, 2013b, 2010; Kobayashi et al. 2015; Wong and Blobel 2008; Wong et al. 2006; Nakano et al. 2010). The dysfunctional operation of Nups and NPCs has been linked to autoimmune disorders(Jühlen and Fahrenkrog 2023), viral infections(Dharan and Campbell 2024), neurological conditions (Chou et al. 2018; Zhang et al. 2020), cardiomyopathies(Burdine et al. 2020), and malignancies, including leukemia (Wong 2021; Jahangiri 2024; Yang et al. 2023; Ikliptikawati et al. 2024). However, traditional imaging techniques have been limited in capturing these dynamic changes, as they primarily provide static snapshots.

Recent breakthroughs in cryo-electron microscopy, machine learning-based predictions, and biochemical reconstitution have provided near-atomic resolution of the NPC linker scaffold within the central core (Rush et al. 2023; Mosalaganti et al. 2022; Petrovic et al. 2022; Bley et al. 2022; Singh et al. 2024). These methods have detailed nucleoporin complexes and their interactions within the scaffold core. However, the techniques for real-time nanoscale imaging of NPC dynamics remain limited. Optical imaging lacks the resolution to observe subcellular structures, while cryo-EM and super-resolution microscopy, though overcoming diffraction limits, primarily rely on fixed samples, raising concerns about their ability to capture true dynamic processes in living cells.

HS-AFM has revolutionized NPC research by allowing real-time visualization of its dynamic structure and transport mechanisms at nanometer resolution. HS-AFM has directly observed the flexible and adaptive behavior of FG-Nups within the NPC, leading to the “spider cobweb model,” which describes FG-Nup filaments as highly dynamic structures that extend, retract and form transient knots to regulate cargo passage (Mohamed et al. 2017). This real-time observation challenges previous static models and highlights the fluidity of NPC transport dynamics, particularly under pathological conditions such as cancer.

## HS-AFM studies of NPC architecture

Traditional atomic force microscopy (AFM) techniques, which often use Xenopus egg nuclear pores, have been limited to producing static images and measuring stiffness at relatively slow speeds(Kramer et al. 2008; Bestembayeva et al. 2015). These methods also lack the necessary spatial resolution to probe the core channel of FG-Nups. While Xenopus oocytes are an excellent model for studying nucleocytoplasmic transport due to their large size (1000 μm) (Sakiyama et al. 2016) compared to mammalian nuclei (10–20 μm), facilitating the isolation of nuclear envelopes (NEs), there are key compositional and functional differences in the NPCs between species. Notably, mammalian NPCs, particularly during cell division, nuclear envelope rupture and repair in cancer cell migration, the role of oncogenes and tumor suppressors in viral trafficking, and chromatin modifications, exhibit significant differences compared to Xenopus egg NPCs. Therefore, it is crucial to investigate the spatiotemporal dynamics of mammalian (human) NPCs directly.

We demonstrated that HS-AFM can directly identify nanotopographical alterations of the nuclear pore inner channel in colorectal cancer (CRC) cells. We proposed the “spider cobweb model”, where FG-NUP filaments within the NPC behave like a tangled, flexible cobweb (Mohamed et al. 2017). These filaments extend, retract, and entangle to form transient knots, selectively allowing cargo passage while maintaining a barrier. This dynamic structure resembles spiders maneuvering within a sticky web, a crucial mechanism for regulating transport efficiency and selectivity, particularly in cancer cells where FG-NUP resilience and behavior differ. Our model contrasts with previous NPC transport models (Mallik et al. 2024; Minasbekyan and Badalyan 2023; Rush et al. 2023) by emphasizing the flexibility and dynamic nature of FG-NUPs, especially under pathological conditions like cancer.

HS-AFM’s high temporal resolution captures the fast, dynamic behavior of intrinsically disordered regions (IDRs) of FG-NUPs, which form the NPC’s selective permeability barrier. Studies using HS-AFM have probed NPC function, particularly in colorectal cancer cells (Mohamed et al. 2020, 2017). In normal cells, FG-NUPs within the central channel form a dynamic, writhing spider cobweb network that maintains selective barrier function. In contrast, in cancer cells, FG-NUP filaments become disorganized, thicker, and more prone to knotting, which diminishes NPC resilience and impairs transport regulation.

HS-AFM has also visualized NPC changes during cell death. In colorectal cancer cells treated with MLN8237 (alisertib), significant NPC deformations were observed as cells underwent apoptosis and autophagy. The normally flexible FG-NUP barrier collapses, leading to NPC structural failure. This “dying code” marks the irreversible breakdown of cellular transport processes, characterized by reduced pore diameter, increased filament entanglement, and loss of FG-NUP flexibility.

Beyond static snapshots, HS-AFM has allowed the mechanistic exploration of NPC function by tracking FG-NUP filament movements. In cancer cells, the central plug of the NPC becomes destabilized, resulting in more frequent and uncontrolled pore openings. HS-AFM reveals how the FG-NUP network temporarily opens to accommodate large molecules and rapidly closes to maintain selective permeability, a process essential for cellular homeostasis (Mohamed et al. 2020, 2017).

**Fig. 3:**
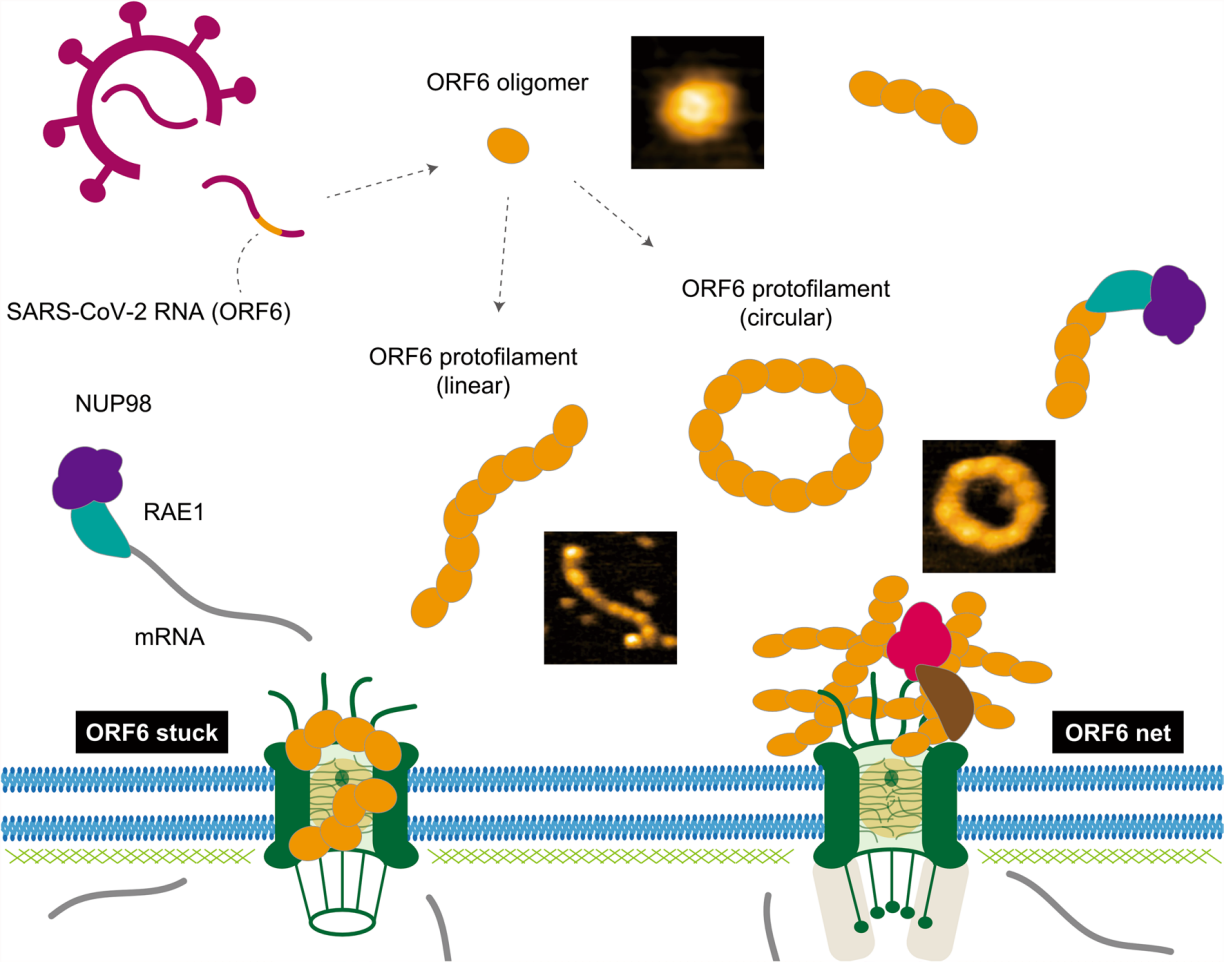
ORF6 effects on NPC function. This figure illustrates how the open reading frame 6 (ORF6), an accessory protein of SARS-CoV-2, self-assembles into aggregates within infected cells. ORF6 amyloidogenic peptides form amyloid fibrils via oligomerization, with the oligomers assembling into protofilaments that adopt circular or linear arrangements. ORF6 directly interacts with the NUP98-RAE1 complex, crucial for mRNA transport, inhibiting its function. Additionally, ORF6 oligomers are hypothesized to disrupt cytoplasm-nucleus transport by either blocking NPCs or forming ordered net-like protofilament structures within the NPC

SARS-CoV-2 ORF6 protein was shown to sequester host nuclear pore proteins, Rae1 and Nup98, in the cytoplasm, inhibiting normal mRNA export, which could play a crucial role in immune evasion (Kato et al. 2021; Miorin et al. 2020; Addetia et al. 2021; Yoo and Mitchison 2024). Nishide et al. used HS-AFM to explore the molecular dynamics of the ORF6 protein, revealing its oligomerization and protofilament formation. The study identified that ORF6 self-assembles through hydrophobic interactions, forming circular or linear protofilaments depending on temperature and lipid substrate conditions (Fig. 3) (Nishide et al. 2023).

This cytoplasmic retention of nuclear pore proteins disrupts nucleocytoplasmic transport, effectively blocking host immune responses, especially interferon signaling. ORF6’s amyloidogenic properties, supported by its spontaneous filament formation, suggest that it could cause cellular dysfunction and contribute to amyloid-related complications seen in COVID-19 patients. The protein’s aggregation was influenced by temperature, with high fever conditions accelerating filament formation, which could exacerbate disease progression. This study enhances the understanding of ORF6’s role in viral immune suppression and its potential contribution to severe disease outcomes through aggregation and interference with nuclear transport (Nishide et al. 2023).

We have developed a rapid strainer microfiltration (RSM) method for isolating nuclei from mouse brain tissue, enabling real-time HS-AFM imaging of NPCs. This approach involves mechanical homogenization followed by sequential filtration, preserving NPC biological activity without fixation (Qiu et al. 2024). The nuclei are immobilized on poly-L-lysine-coated mica and scanned using HS-AFM, capturing dynamic FG-NUP behavior in unprecedented detail. The method confirms octameric ring structures and FG-NUP conformational changes, resembling a “spider cobweb” (Qiu et al. 2024). These findings provide critical insights into NPC function and its role in diseases like cancer and neurodegeneration.

In summary, HS-AFM has advanced our understanding of NPC architecture and function beyond static models, providing a real-time perspective on FG-Nup dynamics, NPC remodeling in disease, and viral nuclear entry mechanisms. These findings establish HS-AFM as an indispensable tool for studying nuclear pore dynamics and their implications in health and disease.

## HS-AFM in chromatin and nucleosome research

Chromatin organization is essential for maintaining genome integrity and regulating gene expression (Fursova and Larson 2024; Henninger and Young 2024; Downs and Gasser 2024). The chromatin remodelers modify chromatin structure, ensuring that the genome remains responsive to cellular signals(Wong and Tremethick 2024). HS-AFM has provided valuable insights into the dynamic organization of chromatin in relation to NPCs, revealing how chromatin is continuously remodeled in response to transcriptional activation or DNA damage.

**Fig. 4:**
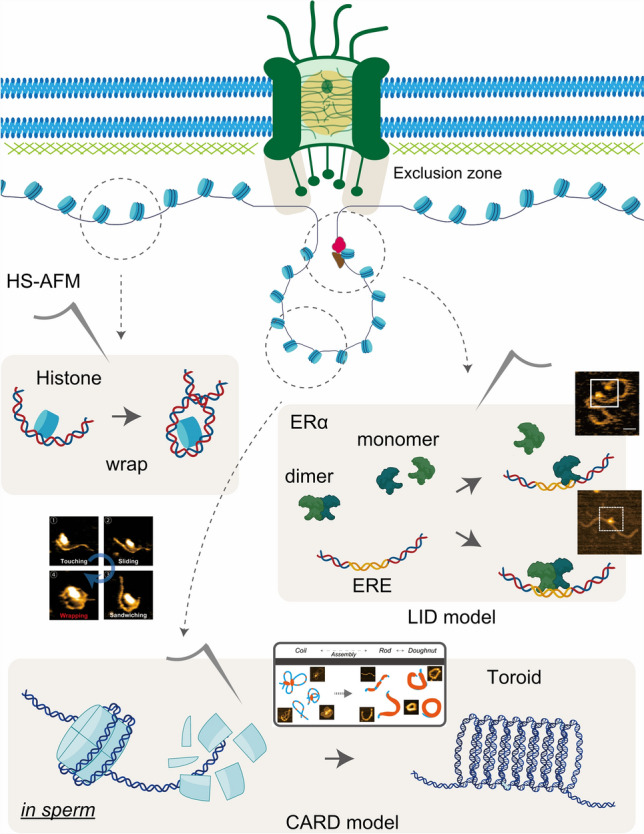
Chromatin remodeling dynamics near nuclear pores observed by HS-AFM. This schematic illustrates dynamic chromatin processes occurring in proximity to nuclear pore complexes (NPCs), as visualized using HS-AFM. While NPCs are classically known for their role in molecular transport between the nucleus and cytoplasm, emerging HS-AFM data reveal chromatin remodeling events that frequently localize near NPCs. These include nucleosome unwrapping, chromatin compaction transitions, and the search behaviors of transcription factors. Notably, in spermatids, intermediate stages of nuclear chromatin condensation—such as histone-to-protamine exchange and toroid-like chromatin structuring—remain poorly understood, yet are now becoming observable with HS-AFM. The CARD (Coil-Assembly-Rod-Doughnut) model is proposed as a conceptual framework for understanding how chromatin dynamics may be modulated at the nuclear periphery. Additionally, the LID (Ligand-Induced Dimerization) model, derived from HS-AFM studies of estrogen receptor α (ERα), provides insight into how ligand binding enhances transcription factor specificity and DNA engagement. Together, these observations highlight the utility of HS-AFM in capturing dynamic chromatin behaviors near NPCs, opening new avenues for dissecting nuclear organization and gene regulation

Nucleosomes are the fundamental units of chromatin, consisting of DNA wrapped around histone proteins. Histones, such as H2A, H2B, H3, and H4, play critical roles in regulating DNA accessibility, influencing processes such as transcription, replication, and DNA repair (Liu et al. 2024; Sato et al. 2022) (Fig. 4). Understanding the dynamic behavior of nucleosomes is essential to unraveling chromatin regulation. Traditional imaging techniques, while informative, lack the spatiotemporal resolution needed to capture rapid nucleosome dynamics. HS-AFM has emerged as a cutting-edge tool to visualize these processes in real-time, allowing researchers to observe nucleosome conformational changes and DNA interactions with unprecedented detail.

Nishide et al. (2021) utilized HS-AFM to explore how the histone protein H2A interacts with DNA, revealing critical insights into nucleosome dynamics and their impact on gene regulation. This study highlighted the ability of HS-AFM to visualize the dynamic interaction between H2A and short double-stranded DNA (dsDNA). The real-time imaging captured an inchworm-like motion of DNA as it wrapped around H2A on lipid substrates, a phenomenon not observed on more static substrates like poly-L-lysine. The interaction between DNA and H2A was highly dependent on the substrate used for imaging, suggesting that the local environment plays a pivotal role in nucleosome behavior. HS-AFM for the first time provided the necessary temporal resolution to observe the formation and dissociation of DNA-histone complexes, demonstrating the potential of this technique to explore the epigenetic regulation of gene expression through nucleosome positioning (Nishide et al. 2021).

Later, Morioka et al. (2023) also explored the role of the histone variant H2A.Z in nucleosome dynamics. H2A.Z is known for its involvement in gene regulation, and this study demonstrated that nucleosomes containing H2A.Z exhibit spontaneous sliding along DNA, a key mechanism for exposing regulatory sequences during transcription. HS-AFM enabled the direct visualization of these sliding events, which occurred over subsecond timescales. The study found that H2A.Z nucleosomes are more unstable than canonical H2A nucleosomes, which facilitates their movement along the DNA strand. This dynamic behavior likely plays a critical role in chromatin remodeling, allowing transcription factors and other regulatory proteins access to the DNA. By quantifying the frequency and magnitude of nucleosome sliding, the authors were able to establish a molecular basis for the role of H2A.Z in gene activation and repression (Morioka et al. 2023).

More recently, Filliaux et al. (2024) focused on centromere nucleosomes, specifically comparing canonical H3 nucleosomes with CENP-A nucleosomes. The centromeres are essential for chromosome segregation during cell division and CENP-A plays a key role in maintaining centromere function. Using HS-AFM, the authors were able to visualize spontaneous unwrapping and rewrapping of DNA around both H3 and CENP-A nucleosomes. Interestingly, CENP-A nucleosomes exhibited greater stability than H3 nucleosomes, which may be critical for ensuring the structural integrity of centromeres during mitosis. This study provided new insights into how the unique structural properties of CENP-A contribute to centromere function. The ability to visualize nucleosome dynamics in real-time allowed the authors to explore the mechanisms underlying centromere stability and the role of nucleosome positioning in chromatin architecture (Filliaux et al. 2024).

HS-AFM has the potential to revolutionize the study of chromatin architecture and gene regulation. One key challenge in chromatin biology is understanding the role of intrinsically disordered regions (IDRs) in transcriptional regulation. For example, protamines (PRMs) play a crucial role in sperm chromatin condensation, replacing histones to form PRM-DNA complexes. However, the precise mechanisms of PRM-mediated DNA condensation remain poorly understood. Using HS-AFM, we directly visualized the real-time binding dynamics of protamine to DNA under physiological conditions. Our observations reveal that protamine insertion initiates DNA coiling, with a heterogeneous spatial distribution of PRM-induced looping. As PRM levels increase, DNA undergoes progressive folding transitions, forming coiled-like structures that evolve into clockwise spirals, rod-shaped intermediates, and ultimately toroid-like nanostructure. Based on these findings, we propose the CARD (coil-assembly-rod-doughnut) model, describing the stepwise process of toroid formation during DNA condensation. This study highlights the versatility of HS-AFM in capturing PRM-DNA interactions in real time, offering critical insights into sperm chromatin architecture. Our results provide a foundation for future research into chromatin organization, reproductive biology, and nucleic acid therapeutics (Nishide et al. 2025a) (Fig. 4).

Moreover, estrogen receptor alpha (ERα) interacts with estrogen response elements (EREs) to regulate gene expression, yet the molecular basis of its ligand-independent binding remains unclear (Klinge 2001) (Fig. 4). ERα plays a central role in controlling gene activity, especially in cancers that respond to estrogen, such as many types of breast cancer. Despite its importance, the full structure and behavior of ERα at the molecular level have remained unclear. HS-AFM, with improved molecular tracking algorithms and enhanced feedback control systems, could provide real-time visualization of ERα–DNA interactions, offering new insights into hormone-independent transcriptional regulation in cancer biology.  In our study, we used HS-AFM to observe how ERα binds to specific DNA sequences EREs 2025). Surprisingly, we found that ERα can attach to DNA even without estrogen, but the presence of estrogen significantly improves the accuracy and stability of this binding. We also captured the structural shift of ERα from a single molecule to a paired (dimerized) form, which appears to be key for its precise DNA targeting. From these results, we propose a new model called “Ligand-Induced Dimerization” (LID), in which estrogen triggers ERα to pair up and efficiently bind DNA. This discovery sheds new light on how hormone signals control genes and may help guide the development of future cancer treatments. (Nishide et al. 2025b) (Fig. 4) 

HS-AFM has revolutionized the study of nucleosome dynamics by providing real-time, high-resolution imaging of histone-DNA interactions. These three studies collectively demonstrate the versatility of HS-AFM in exploring different aspects of nucleosome behavior, from histone variant dynamics to the structural stability of centromere nucleosomes. By capturing the rapid conformational changes of nucleosomes, HS-AFM provides a powerful tool for understanding the fundamental processes governing chromatin regulation, with implications for gene expression, DNA repair, and chromosome segregation.

## Future directions

Despite the remarkable advancements achieved with HS-AFM, several key questions remain regarding NPC and chromatin dynamics, particularly in the context of viral invasion, gene regulation, and chromatin remodeling. Recent studies suggest that HIV-1 capsid entry into the nucleus is facilitated by direct interactions with nucleoporins such as Nup153 and Nup358, bypassing traditional nuclear transport pathways through phase partitioning within the NPC (Lim et al. 2024). The structural analyses have revealed that the cone-shaped HIV-1 capsid induces mechanical stress on NPC scaffold rings, leading to structural cracks that widen the nuclear pore (Kreysing et al. 2025). This mechanism is critical for understanding how HIV-1 infects non-dividing cells and evades immune detection, yet many aspects of this process remain elusive. HS-AFM’s ability to capture real-time, nanometer-scale molecular interactions presents a unique opportunity to resolve the precise conformational changes of FG-Nups during capsid translocation, providing direct evidence of NPC widening and capsid uncoating dynamics. Future studies could employ HS-AFM combined with single-molecule tracking techniques to visualize capsid-NPC interactions in physiologically relevant conditions, shedding light on how host nuclear pore proteins and immune factors modulate HIV nuclear entry. Additionally, HS-AFM could be used to assess the efficacy of antiviral drugs like GS-6207, which target capsid stability, in real-time, offering mechanistic insights into viral persistence and immune evasion strategies.

**Fig. 5:**
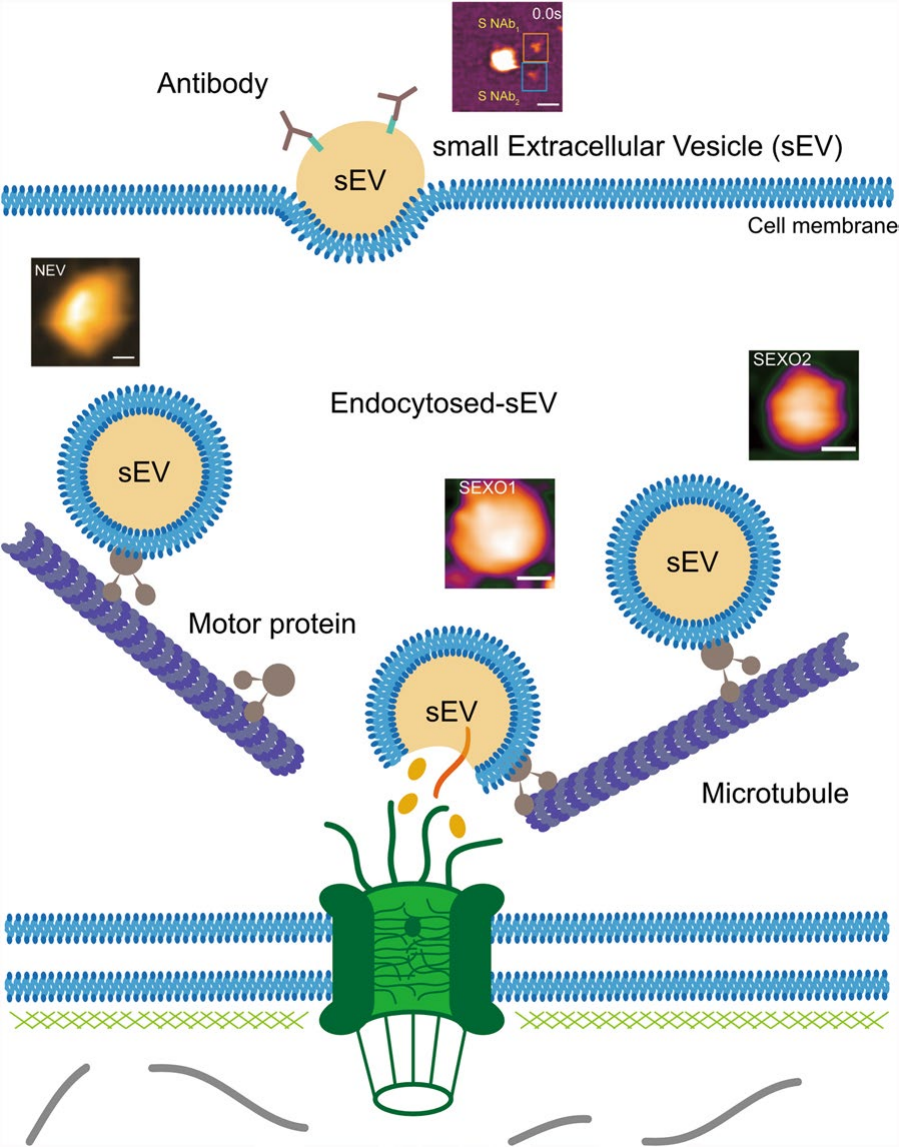
Extracellular vesicle (EV) transport toward the NPC and its role in chromatin regulation. EVs are transported along the microtubule network by motor proteins such as dynein and kinesin, directing them toward the nuclear envelope. Upon reaching the NPC, EVs can dock, fuse, or release their cargo into the perinuclear space or directly into the nucleus. The released cargo, including non-coding RNAs, transcription factors, and chromatin-associated proteins, may contribute to chromatin remodeling, gene regulation, and epigenetic modifications. This process plays a potential role in transcriptional reprogramming, cellular differentiation, and disease progression. Future studies integrating HS-AFM and live-cell imaging will help elucidate the spatiotemporal dynamics of EV-mediated chromatin modifications and their impact on nuclear function

Interestingly, extracellular vesicles (EVs) play a crucial role in intercellular communication, delivering proteins, RNAs, and other bioactive molecules to target cells (Sandira et al. 2025; Sajidah et al. 2024, 2022). Recent studies suggest that EVs utilize the microtubule network and motor proteins, such as dynein and kinesin (Guillaud et al. 2008; Wong et al. 2002; Hirokawa et al. 2006; Hirokawa 1998), for directed transport toward the nuclear envelope. Once near the NPC, EVs may dock, fuse, or release their cargo into the perinuclear space, facilitating the transfer of regulatory molecules directly into the nucleus. This mechanism could be essential for modulating gene expression, chromatin remodeling, and viral genome delivery, with potential implications in cancer progression, immune regulation, and neurodegenerative diseases. Notably, EV-derived non-coding RNAs and chromatin-associated proteins have been implicated in epigenetic modifications, suggesting a potential role for EV-mediated chromatin remodeling in transcriptional regulation and cellular reprogramming. Future studies integrating HS-AFM with live-cell imaging could provide real-time insights into EV-NPC interactions and their impact on chromatin architecture, uncovering the molecular basis of nuclear-targeted vesicular transport (Fig. 5).

## Technical advancements required for HS-AFM to further explore NPC and chromatin dynamics

To fully unravel the complex dynamics of NPCs and chromatin, several technical advancements in HS-AFM are required to enhance its temporal resolution, spatial accuracy, imaging depth, and data analysis capabilities. One of the most critical improvements needed is higher temporal resolution to capture ultrafast biomolecular interactions, such as NPC conformational fluctuations during nucleocytoplasmic transport. While current HS-AFM imaging speeds (~ 100 ms per frame) already provide exceptional temporal resolution, further enhancements are essential for observing rapid molecular rearrangements with even greater precision. To achieve this, adaptive scanning algorithms powered by artificial intelligence (AI) could dynamically adjust scanning rates based on molecular motion, ensuring that transient structural changes are accurately recorded in real time.

Another key area of advancement is enhanced spatial resolution with functional imaging. Although HS-AFM achieves sub-nanometer lateral resolution, resolving individual nucleoporin movements within the NPC core remains challenging. The improvements in finer tip geometries and multi-angle imaging capabilities would enable more precise tracking of nucleoporin interactions during nuclear transport. Additionally, the integration of correlative HS-AFM with super-resolution fluorescence microscopy would allow simultaneous visualization of NPC conformational changes and cargo interactions, providing a more comprehensive understanding of how NPCs regulate molecular transport and genome organization.

To extend HS-AFM’s applicability to chromatin research, innovations in imaging depth and three-dimensional (3D) HS-AFM are required. Currently, HS-AFM is primarily limited to surface structures, making it difficult to study deep chromatin organization within the nucleus. Developing 3D HS-AFM with controlled force spectroscopy could enable layer-by-layer imaging of chromatin compaction and NPC structural changes in response to transcriptional activity, stress, or viral invasion. Such advancements would provide unprecedented insights into how chromatin architecture influences nuclear function in both healthy and diseased states.

A major limitation of current HS-AFM techniques is that most experiments are conducted on isolated nuclei or reconstituted systems, which may not fully capture native NPC and chromatin dynamics. To overcome this, non-invasive, long-term imaging of live cells must be developed. Innovations in low-force scanning and temperature-controlled liquid cells could facilitate continuous HS-AFM imaging of live cells, preserving the physiological integrity of NPC-chromatin interactions within intact nuclear membranes. This would allow researchers to study real-time chromatin remodeling, nucleocytoplasmic transport, and viral-host interactions in their native environments.

Finally, AI-driven image processing for high-throughput analysis will be essential for handling the vast amounts of nanoscopic data generated by HS-AFM. Automated AI-based image segmentation and classification would enable precise tracking of nucleoporin dynamics, chromatin remodeling events, and viral capsid interactions with NPCs. Furthermore, the machine learning models trained on HS-AFM datasets could enhance pattern recognition in nuclear transport dynamics, facilitating the automated identification of structural abnormalities associated with disease states, such as cancer, neurodegeneration, and viral infections.

To gain a comprehensive understanding of NPC function and chromatin regulation, HS-AFM must be integrated with other advanced imaging modalities. Combining HS-AFM with cryo-electron tomography, super-resolution microscopy, and single-molecule tracking would bridge nanoscale dynamics with cellular-scale processes, offering unprecedented insights into nuclear organization and gene regulation. Furthermore, the integration of AI-enhanced predictive modeling could enable in silico simulations of NPC transport and chromatin folding, complementing experimental HS-AFM observations.

## Conclusion

HS-AFM has transformed our ability to study the real-time dynamics of nuclear pore complexes and chromatin, revealing novel insights into nucleocytoplasmic transport, transcriptional regulation, and viral nuclear entry. With ongoing technical advancements—including higher temporal resolution, enhanced spatial imaging, AI-driven analysis, and live-cell compatibility—HS-AFM is poised to further unravel the complexity of NPC function and chromatin remodeling. These innovations will be crucial in understanding diseases linked to nuclear transport dysfunction, such as cancer, neurodegeneration, and viral infections, ultimately paving the way for new diagnostic and therapeutic strategies.

## Data Availability

Not applicable.
